# Determinants of hyena participation in risky collective action

**DOI:** 10.1098/rspb.2023.1390

**Published:** 2023-11-29

**Authors:** Tracy M. Montgomery, Kenna D. S. Lehmann, Samantha Gregg, Kathleen Keyser, Leah E. McTigue, Jacinta C. Beehner, Kay E. Holekamp

**Affiliations:** ^1^ Department of Integrative Biology and Program in Ecology, Evolution, and behavior, Michigan State University, 288 Farm Lane, East Lansing, MI 48824, USA; ^2^ Human Biology Program, Michigan State University, 288 Farm Lane, East Lansing, MI 48824, USA; ^3^ Mara Hyena Project, PO Box 164-00502, Karen, Nairobi, Kenya; ^4^ Department for the Ecology of Animal Societies, Max Planck Institute for Animal Behavior, Bücklestraße 5a, 78467 Konstanz, Germany; ^5^ Center for the Advanced Study of Collective Behavior, University of Konstanz, Universitätsstraße 10, 78464 Konstanz, Germany; ^6^ Rocky Mountain Research Station, Colorado State University, 240 W Prospect St, Fort Collins, CO 80525, USA; ^7^ Department of Psychology, University of Michigan, 530 Church Street, Ann Arbor, MI 48109, USA; ^8^ Department of Anthropology, University of Michigan, 1085 South University Avenue, Ann Arbor, MI 48109, USA

**Keywords:** cooperation, *Crocuta crocuta*, interspecific competition, lions, mobbing, spotted hyenas

## Abstract

Collective action problems arise when cooperating individuals suffer costs of cooperation, while the benefits of cooperation are received by both cooperators and defectors. We address this problem using data from spotted hyenas fighting with lions. Lions are much larger and kill many hyenas, so these fights require cooperative mobbing by hyenas for them to succeed. We identify factors that predict when hyena groups engage in cooperative fights with lions, which individuals choose to participate and how the benefits of victory are distributed among cooperators and non-cooperators. We find that cooperative mobbing is better predicted by lower costs (no male lions, more hyenas) than higher benefits (need for food). Individual participation is facilitated by social factors, both over the long term (close kin, social bond strength) and the short term (greeting interactions prior to cooperation). Finally, we find some direct benefits of participation: after cooperation, participants were more likely to feed at contested carcasses than non-participants. Overall, these results are consistent with the hypothesis that, when animals face dangerous cooperative dilemmas, selection favours flexible strategies that are sensitive to dynamic factors emerging over multiple time scales.

## 1. Introduction

Humans and other animals are predicted to cooperate when the net benefits of cooperation exceed benefits accruing to individuals acting alone [1]. One type of cooperation is collective action, where many individuals cooperate to gain group-level benefits [2]. In animals, collective action includes both intra- and inter-specific conflicts, such as driving away predators or competitors, and defending territory, offspring, or resources [3]. The group-level cooperation that occurs during collective action is an emergent property of decisions made by individuals to cooperate or defect [4,5]. Collective action problems arise when group members choose to pursue individual rather than group benefits; where defectors are able to enjoy the collective benefits of cooperators, ‘cheater’ strategies can arise [2].

What drives an individual to cooperate, rather than defect, when faced with a collective action problem? Participation in collective action can yield important individual-level benefits, including: acquisition or defense of resources [1], kin-selected fitness benefits among highly related group members [6], and/or other indirect benefits and social incentives, such as an enhanced reputation with potential coalition partners or mates [7,8]. However, participation is usually costly, involving opportunity and energetic costs, and risk of injury or death [9,10].

Previous work has predominantly focused on collective action in homogeneous animal groups [11], but individual and relational heterogeneity in social groups can strongly influence decisions regarding whether or not to participate in collective action [12,13]. Theoretical modelling suggests that group members are most likely to participate when they can expect the biggest share of the benefits or rewards, can contribute for the lowest cost, or are the most capable (e.g. largest, strongest) [14,15]. Other theoretical studies have demonstrated the importance of social network connections to successful collective action, especially in societies where social relationships are critical to fitness [16,17]. However, empirical studies about collective action within heterogeneous animal groups are rare. Furthermore, although we have developed a deep understanding of how cooperation and collective action can evolve, some forms of cooperative behaviour have received much more attention than others. The mobbing of predators or competitors represents a crucial facet of cooperative animal behaviour [3,9], yet it is often underemphasized when compared to other behaviours such as alloparental care, cooperative hunting and intergroup conflict.

Spotted hyenas (*Crocuta crocuta*) are an ideal study system in which to investigate collective action in heterogeneous social groups: they live in complex, differentiated societies, called clans, which are large (≤130 individuals), mixed-sex, fission–fusion groups [18]. All clan members know one another individually, rear their cubs together at a communal den, and defend a common territory [19], but to avoid competition, clan-mates spend much of their time alone or in small subgroups [20]. Due to female philopatry and male dispersal, most east African clans are composed of multiple matrilines of adult females, their offspring, and several adult immigrant males [21]. Mean relatedness among clan-mates is very low: mean *R*-values are no higher among natal animals (0.011 ± 0.002) than among immigrant animals who arrive from multiple neighboring clans (0.009 ± 0.007) [22]. Each clan is structured by a strict linear dominance hierarchy, with natal animals outranking immigrants [19]. Social rank has large fitness effects because it allows high-ranking group members to usurp food from clan-mates [23,24], and food access strongly affects reproductive success among females [25].

**Figure 1.**
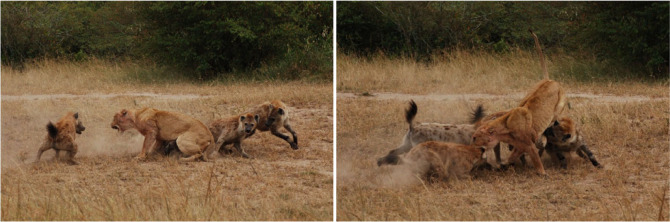
A group of four hyenas mobbing a lioness. Photos by Brittany Gunther.

Hyena clan-mates frequently cooperate during collective action in diverse contexts [26], including the collective mobbing of lions (*Panthera leo*; figure 1). Cooperative mobbing is a conspicuous example of collective action, which occurs when two or more individuals synchronously approach or attack a threat [27]. Lions are spotted hyenas' main competitors; these species use the same food resources and frequently kleptoparasitize one another [28]. By mobbing lions, hyenas can overwhelm them and drive them away [19], increasing hyenas’ probability of feeding when competing with lions over food [29]. Lions are larger and stronger than hyenas (2.4 times larger by mass) [30] and represent a main source of mortality among hyenas [28], with at least 27% of hyena deaths with known causes attributed to lions in this population [31]. Mobbing lions is, therefore, very risky for hyenas, and—as it often results in benefits to both cooperating and defecting group members [8,32]—mobbing represents exactly the conditions under which cheating is expected to destabilize cooperation [2,3].

Here, we aim to identify the mechanisms that drive cooperation in a complex society characterized by differentiated social relationships based on kinship, sex, age and social rank. We focus on the collective mobbing of lions by wild spotted hyenas in Kenya, and use a detailed, long-term dataset to investigate when cooperative mobbing occurs, who participates in cooperative mobbing and who benefits from it.

With respect to when mobbing occurs, based on past theoretical and empirical studies of inter-group conflict [33], we expected that both relative group size, and the ecological and social context in which lions and hyenas interact, would be critical variables. Specifically, we predicted that hyenas would be more likely to mob lions at valuable resources, such as the communal den or carcasses, especially when prey are scarce [29,34]. We also predicted that hyenas would be more likely to mob lions when risks to individual hyenas are lower; namely, when male lions are absent and when the ratio of lions to hyenas is lower [35,36]. Finally, we predicted that groups of hyenas would be more likely to mob together when they engage in affiliative interactions with group-mates or when they are more closely associated with the other individuals present [37,38].

With respect to who participates in mobbing, based on the theoretical modeling of Gavrilets and colleagues [14,15], we predicted that participants would be those with the lowest cost-benefit ratios. Hyenas would be more likely to participate, and less likely to defect, when they are high-ranking and thus have priority of access to any resources obtained via mobbing [15,39]. We also predicted that hyenas would be more likely to participate when they are in top physical condition (i.e. prime-aged and good nutritional state), such that they can escape from lions more easily and thus bear a lower cost of participation [14,40]. Based on theoretical studies showing the importance of social networks to successful cooperation [16,17], we also predicted that hyenas would be more likely to participate when their social allies or kin are present, as occurs in other socially complex species [41,42]. Finally, hyenas engage in ritualized greeting behaviour, which functions to promote cooperation and reinforce social bonds [43]; we thus expected that occurrence of this affiliative behaviour shortly before mobbing would increase an individual's likelihood of participating [38,44].

With respect to who benefits from cooperative mobbing, we focused on potential individual-level food resource benefits of mobbing [29]. We predicted that mobs would be more likely to occur when higher quality and/or larger food items are present [45]. We also predicted that hungrier hyenas, as reflected by belly size, would be more likely to participate in mobbing when food is present [46]. Most importantly, we predicted that hyenas who participate in cooperative mobbing would be more likely to obtain food [29].

## 2. Methods

From 1988–2016, we monitored four clans of wild spotted hyenas in the Maasai Mara National Reserve, Kenya. We observed one clan from 1988–2016 and three clans from 2008–2016. We monitored clans daily during two observation periods, from 05.30 to 10.30 and from 16.00 to 21.00. When we encountered a subgroup of one or more hyenas, we initiated an observation session (session) and recorded the identities of all hyenas present within a 200 m radius, using their unique spot patterns and ear damage to recognize individuals. We also recorded the number, sex, and age class of all lions found [47]. Sessions lasted from 5 min to several hours and ended when behavioural interactions ceased, and observers left that individual or group. Using all-occurrence sampling [48], we recorded arrivals and departures of individual hyenas, agonistic interactions, and greetings. Greetings are affiliative interactions occurring when two partners stand parallel to one another but facing in opposite directions to sniff the other's anogenital region [19]. We also performed scan-sampling [48] every 20 min throughout each session to document change in hyenas present.

In our population, lions and hyenas co-occurred in an average of 4 sessions per clan per month, and the two species interacted by directing behaviour at one another in 44% of those co-occurrence sessions [29,49]. Throughout each session involving both lions and hyenas, we recorded all mobbing events using all-occurrence sampling. We operationally defined ‘mobbing’ as a group of two or more hyenas, usually side-by-side and within 1 m of one another, with tails bristled over their backs, approaching within 10 m of at least one lion (figure 1) [29]. In association with each mobbing event, we recorded the identities of all participating hyenas, and the number, sex and age class of the lions being approached.

Throughout each lion–hyena session in which a kill or carcass was present, we recorded hyena feeding behaviour. Because lion–hyena sessions are often very chaotic (and thus the ability of the observer to record feeding behaviour varies), we created a simple feeding dataset of one-zero sampling for each hyena present at each session. For each minute of each session, we recorded whether or not a focal hyena was observed feeding.

Because hyena societies are fission–fusion and most individuals spend the majority of their time alone or in small subgroups, we measured the strength of social relationships among individuals by calculating association indices [50]. Simple ratio association indices [51] were calculated for each dyad in each session using R package *asnipe* [52] based on patterns of association over the previous 365 days.

### (a) When does cooperative mobbing occur?

Here, we restricted our dataset to observation sessions where lions and hyenas interacted. We operationally defined interspecific interactions as occurring when lions and hyenas directed behaviour at one another or when lions and hyenas approached within 10 m of one another [29]. We further filtered to sessions with field notes of high-enough quality to be certain that all mobbing events were recorded if they were observed. Finally, we excluded sessions where only one hyena was present because, by definition, multiple hyenas are required for a mob to occur. We fitted a logistic regression where our response variable was binomial: whether or not a mob occurred during that session. Fixed effect covariates included key environmental and contextual variables with the potential to affect mobbing occurrence (table 1; Model A in electronic supplementary material, table S1). We included interactions between session length and the number of hyenas present, and between session length and the number of hyenas that greet (greeters), to control for the possible correlation between observation time and number of hyenas or greetings observed. We included interactions between number of hyenas present and total number of lions present, and between number of hyenas present and male lions present based on past work indicating that the ratio of lions to hyenas can affect mobbing behaviour [35,36]. We included interactions between hyena and lion variables (number of hyenas present, number of lions present, male lions present) and social variables (number of greeters, mean association index) to investigate whether social behaviour could help overcome the barriers to mobbing we documented earlier [29]. No random effects were included in this model; clan was considered as a random intercept but was dropped as it explained no variance.

**Table 1.** Observation session- and individual-level predictors used in models of mobbing behaviour (see ‘Model parameters' in electronic supplementary material for more details about each variable).

observation session variables		
variable	values	details
session length	number of minutes	total duration of observation session
session context	food, den, other	describes if session occurs near a kill, a hyena den, or neither
prey density	monthly prey density for each clan	measured as standard deviations above or below yearly mean prey density based on biweekly census transects
number of hyenas present	count of individuals	total number of hyenas present
number of lions present	count of individuals	total number of lions present
male lions present	yes, no	presence/absence of adult male lions
number of male lions present	count of individuals	total number of adult male lions present
number of hyenas who greet (greeters)	count of individuals	total number of hyenas who engaged in greeting behaviour during the session
mean association index	ranges from 0 to 1	mean of all dyadic association indices among hyenas present
carcass freshness	fresh, old	fresh is < 24 h old
carcass size	small, medium, large, extra-large	size categories determined by prey species and age (electronic supplementary material, table S5)

individual variables		
variable	values	details
age	years of age	based on appearance when first seen or patterns of teeth wear
sex	male, female	
social rank	ranges from −1 to 1	position in dominance hierarchy based on submissive behaviour
reproductive state (females)	nulliparous, pregnant, lactating, other	calculated for females based on observations of maternal behaviour
dispersal status (males)	immigrant, natal	only calculated for males because females are philopatric
greeted	yes, no	yes if the individual greeted in the 5 min prior to the mob
association index with participants	ranges from 0 to 1	mean of dyadic association indices with mobbing individuals
maternal relatedness with participants	ranges from 0 to 1	proportion of mobbing individuals who were either mother, offspring, or sibling
belly size	gaunt, normal, fat, obese	belly size upon first sighting at observation session

### (b) Who participates in cooperative mobbing?

Here, we restricted our dataset to observation sessions where mobbing occurred and where the identities of more than 90% of mobbing participants were known. For each mob during these sessions, we determined which hyenas were present when the mob occurred based on the arrival and departure times of all hyenas in the session. Each focal hyena present during a mobbing event was coded as either a participant (participant) or non-participant (defector) for that particular mobbing event. We then assigned relevant demographic, physiological, and social variables to each focal hyena: we assigned an age, social rank, reproductive state (females), and dispersal status (males) to each focal hyena present (table 1). We also assigned social context measures to each focal hyena present, including whether or not the focal hyena had greeted in the 5 min prior to a mob (greeted), the average association index between the focal hyena and other participants (association index), and the proportion of participants to which the focal hyena was closely related (i.e. mother, offspring or sibling of the focal hyena; ‘maternal relatedness'; table 1).

To investigate hyena participation in cooperative mobbing events, we fitted a series of logistic mixed-effect models where our response variable was binomial: whether or not the focal hyena participated in that mob. Fixed effect covariates included key demographic and social variables with the potential to affect mobbing participation (Models D-H in electronic supplementary material, table S2). All models included random intercept covariates of hyena identity and of mob nested within session. Clan was not included as a random intercept because it explained only 2.2% of the variance in participation (intraclass correlation coefficient = 0.022).

We built a series of logistic mixed-effect models to investigate the effects of different variable sets on specific categories of hyenas.

#### (i) Preliminary analysis of all hyenas

The first model (Model D in electronic supplementary material, table S2) included all hyenas and included age and sex to identify broad differences between age and sex classes.

#### (ii) Female participation model

The female model (Model F in electronic supplementary material, table S2) was restricted to all adult females (age > 2 years) and included key demographic and social factors with the potential to affect mobbing participation in adult females. We included interactions between social rank and other variables because social rank critically structures hyena social relationships [53].

#### (iii) Male participation model

The male model (Model G in electronic supplementary material, table S2) was restricted to all adult males (age > 2 years) and likewise included key demographic and social factors with the potential to affect mobbing participation in adult males. We included interactions between social rank and other variables. We were not able to include the term for maternal relatedness in this model because many of these individuals were immigrant males for which we do not currently have relatedness data. We were also unable to include an interaction between age and social rank due to its collinearity with social rank.

#### (iv) Juvenile participation model

The juvenile model (Model H in electronic supplementary material, table S2) was restricted to all juveniles (age < 2 years) and included key demographic and social factors with the potential to affect mobbing participation by juvenile hyenas. We also included three interaction terms, age by sex, age by social rank, and sex by social rank.

To ensure that we were measuring the effect of affiliative social interactions and not just that of social interactions more generally, we re-ran top models that included a term for whether or not a hyena greeted to also include a term for whether or not an individual engaged in an aggressive interaction in the 5 min prior to the mob occurring. Although aggressions occur more frequently than greetings in our dataset, in none of these models was the aggression term included in the top model. However, the affiliative term remained in top models, confirming that our greeting measure captures the effect of affiliation specifically and not of social interactions more generally.

### (c) Who benefits from cooperative mobbing?

To investigate potential resource benefits of mobbing, we fit four logistic mixed-effect models (Models I-L in electronic supplementary material, table S3). For all analyses of resource benefits, we restricted our dataset to observation sessions with food present, and further restricted our participants to focal adult hyenas (age > 2 years), as juvenile resource acquisition and defense are strongly dependent on adult support [54,55]. If hyenas mob to obtain or defend food resources, we predicted that mobs would be more likely to occur at sessions where higher quality (fresher) and/or larger food items were present (Model I in electronic supplementary material, table S3). Here, we modified our global model of the probability of mobbing occurrence (Model A in electronic supplementary material, table S1) by including terms for food quality (carcass freshness) and size (‘carcass size’; table 1).

In our second model (Model J in electronic supplementary material, table S3), we predicted that hyenas that were hungrier, or those in a poorer nutritional state, would be more likely to participate in mobbing at sessions with food. Here, we fit a logistic mixed-effects model with a binomial response variable: whether or not the focal hyena mobbed during the session. We restricted our analysis to focal adult hyenas during sessions in which observers had recorded at least one non-normal belly size to create more even categorical distributions for belly size. This model included the following fixed effects: age, sex, social rank, belly size, carcass freshness and carcass size (table 1). We also included interactions between social rank and belly size and between social rank and carcass size because of the large effect that social rank has on resource acquisition [23].

Lastly, we predicted that hyenas who participate in mobbing would be more likely to obtain food, both immediately after the mob and during the session overall. For these analyses, we restricted our dataset to mobs (Model K in electronic supplementary material, table S3) or sessions (Model L in electronic supplementary material, table S3) where at least one hyena fed, and we coded each hyena present as either a mobbing participant or defector. We built two logistic mixed-effects models to examine these predictions, where the response variable was binomial: whether or not that hyena fed. Both models included the following fixed effects: focal hyena age, sex, social rank, and participant status, carcass freshness and size, and interactions between social rank and participant and between participant and carcass size (table 1). Model K investigated the probability of the hyena getting food within 5 min after the mob and included a fixed effect of whether or not the focal hyena participated in that mob. Here, for each mob, our response variable was whether or not the focal hyena fed in the 5 min following the mob. We removed mob identity as a random effect from the global model (Model K) because it explained no variance. Model L investigated the probability of a hyena getting food during the session overall and included a fixed effect of whether or not the focal hyena mobbed during the session. Here, for each session, our response variable was whether or not the focal hyena fed anytime between the first mobbing event and 30 min after the final mobbing event. We excluded later feeding data to reduce feeding observations due to hyena turnover at the carcass as some hyenas become satiated, and we used 30 min as our cut-off because a group of hyenas can reduce a large carcass to bones in under 30 min [19]. We also removed hyena identity as a random effect from the global model (Model L) because it explained no variance.

### (d) Statistical analysis

All analyses were conducted using R version 4.1.2 and RStudio version 2021.09.0. We first performed data exploration by investigating outliers, distributions and collinearity [56]. We tested all global model predictors for multicollinearity using both correlation coefficients and variance inflation factors (VIFs), and we removed collinear predictors until none were collinear, with all correlation coefficients ≤ 0.7 and all VIFs ≤ 3 [57]. All numeric model predictors were z-score standardized immediately before modeling using the scale function in R to allow comparison of coefficients [57]. We used R package *glmmTMB* [58] to build all models, and we performed model selection on the global model using the dredge function in R package *MuMIn* [59]. The top models, as determined by AIC criteria, are depicted in the figures and tables here and in the electronic supplementary material. All top models were visually inspected to confirm assumptions of multicollinearity, normality of residuals, normality of random effects, heteroscedasticity, and homogeneity of variance using R package *performance* [60] and R package *DHARMa* [61]. We also used R package *DHARMa* to inspect all groups and observations for disproportionate influence in our models, but none warranted exclusion. Between-group comparisons were conducted using Tukey post-hoc tests for multiple comparisons of means in R package *multcomp* [62]. Forest plots were created using R package *sjPlot* [63] and prediction plots were created using the ggpredict function in R package *ggeffects* [64] to obtain predicted values and R package *ggplot2* [65] to create the plots from those values. Wald confidence intervals calculated using R package *glmmTMB* [58] are depicted in the main text and figures, but we also generate and interpret more conservative [66] likelihood profile based confidence intervals using R package *broom.mixed* [67] (electronic supplementary material, table S4).

## 3. Results and discussion

### (a) When does cooperative mobbing occur?

We built a series of logistic regressions modeling the occurrence of mobbing as a function of environmental and contextual factors in 325 lion–hyena interaction sessions. Spotted hyenas mobbed in 41.8% (*n* = 136) of these sessions, with a median of 2 mobs per session (mean 3.1, range 1–40) and a median of 4 hyenas per mob (mean 5.1, range 2–16). A median of 2 lions (mean 3.4, range 1–20) were present at sessions where hyenas did not mob, while a median of 2 lions (mean 3.7, range 1–14) were present at sessions where hyenas did mob. Hyenas mobbed at 44% of interaction sessions at carcasses, 35% of interaction sessions at active dens, and 39% of interaction sessions away from either of these resources.

**Figure 2.**
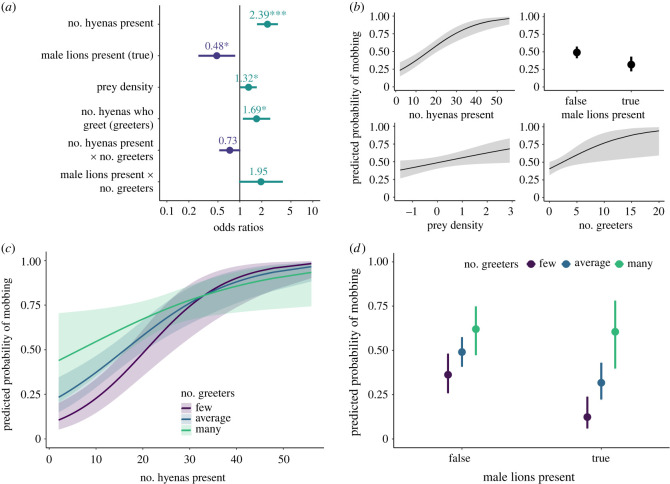
Top model of the predicted probability of mobbing occurrence in sessions where lions and hyenas interact (Model A: *n* sessions = 321). (*a*). Dots depict coefficient estimates, lines depict 95% confidence intervals, and asterisks depict significance at the following *p*-values: * = 0.05; ** = 0.01; *** = 0.001. (*b–d*). Lines (or dots) depict estimated marginal means and shaded areas (or vertical lines) depict 95% confidence intervals. (*c*,*d*). ‘Few’ indicates 1 standard deviation below the mean number of hyenas who greet, ‘average’ indicates the mean number of hyenas who greet, and ‘many’ indicates 1 standard deviation above the mean number of hyenas who greet during sessions in this dataset.

In our model of mobbing occurrence (Model A: *n* = 321 sessions; figure 2; electronic supplementary material, table S4), mobbing was more likely to occur when more hyenas were present (odds ratio (OR) hyenas = 2.39, *p* < 0.001) and when male lions were absent (OR—male lions = 0.48, *p* = 0.014). Counter to our expectations, local prey density was positively correlated with the probability of mobbing (OR—prey = 1.32, *p* = 0.038). Increasing numbers of individuals who engaged in greeting behaviour (greeters) during the session also increased the predicted probability of mobbing (OR—greeters = 1.69, *p* = 0.017). However, a negative but non-significant interaction between number of hyenas present and number of greeters (OR—hyenas×greeters = 0.73, *p* = 0.061) indicated that greetings may facilitate mobbing behaviour when only a few hyenas are present, but may not affect mobbing behaviour when many hyenas are present. A positive but non-significant interaction between male lion presence and number of greeters (OR—male lions×greeters = 1.95, *p* = 0.059) indicated that greetings might have a larger positive effect on mobbing occurrence when male lions are present than when they are absent. Session length, session context, number of lions present, and mean association index of hyenas present were not included in the top model or any model within 6 AIC of the top model.

Overall, our results demonstrate that the decision to mob lions is better predicted by the situational risks of mobbing than the potential benefits: hyenas were most likely to mob in sessions where risk was reduced by more hyenas being present and male lions being absent, regardless of prey abundance or whether there were resources present to defend. Our results indicate that hyenas attend only to the presence or absence of male lions as a source of risk, as they did not otherwise alter their mobbing behaviour based on the number of lions present (Models B and C in electronic supplementary material, table S1 and table S4), suggesting that the ratio of lions to hyenas may be less important than previously thought [35,36]. Finally, we found that greetings were associated with increased mobbing occurrence, particularly when the situational risks were higher (i.e. fewer hyenas or male lions were present). This accords with prior studies suggesting that greetings promote cooperation and reinforce social bonds [43]. Our results imply an additional critical function for greetings as a coordination mechanism allowing hyenas to achieve collective action.

### (b) Who participates in cooperative mobbing?

To understand cooperative mobbing at the individual level, we used logistic regression models to examine the factors predicting an individual's participation in mobbing, given that a mobbing event occurs. This participation dataset consisted of 4740 mob–hyena combinations, with 492 unique hyenas present for 344 total mobs during 119 observation sessions involving lions and hyenas. In 33% (*n* = 1577) of mobbing opportunities, focal hyenas participated in mobs, while in the remaining 67% (*n* = 3163) of mobbing opportunities, focal hyenas were present, but defected. Of the 492 unique hyenas, 44 individuals always mobbed (in a range of 1–5 mobs), and 189 individuals always defected (in a range of 1–44 mobs). The remaining 259 hyenas mobbed in a median of 33% (mean = 38%, range = 2–94%) of mobbing opportunities (median = 9, mean = 14.8, range = 2–94 mobs). Of mobbing participants, 77% were female (23% male) and 89% were adult (11% juvenile); of individuals who were present but did not mob, 57% were female (43% male) and 69% were adult (31% juvenile).

**Figure 3.**
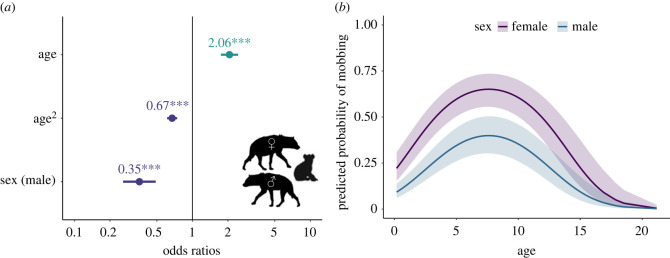
Top model of the predicted probability of mobbing participation by all hyenas (Model D: *n* focal hyenas = 4383; *n* sessions = 117; *n* mobs = 342; *n* unique hyenas = 431). (*a*). Dots depict coefficient estimates, lines depict 95% confidence intervals, and asterisks depict significance at the following p-values: * = 0.05; ** = 0.01; *** = 0.001. (*b*). Lines depict estimated marginal means and shaded areas depict 95% confidence intervals.

In our overall participation model (Model D: *n* = 4383 mob–hyena combinations; figure 3; electronic supplementary material, table S4), females were more likely to mob than males (OR—male = 0.35, *p* < 0.001). Focal individuals of age 7.6 years (range 0.2–21.2 years) were most likely to mob (OR—age = 2.06, *p* < 0.001; OR—age^2^ = 0.67, *p* < 0.001). Because of these clear sex-based differences, which a follow-up model indicated were associated with sex itself and not with sex-related differences in dispersal status or social rank (Model E in electronic supplementary material, table S2 and table S4), we divided all subsequent analyses by sex and age class. Spotted hyenas reach reproductive maturity at 2 years of age [68], so individuals were either juveniles (< 2 years; see electronic supplementary material) or adults (> = 2 years).

**Figure 4.**
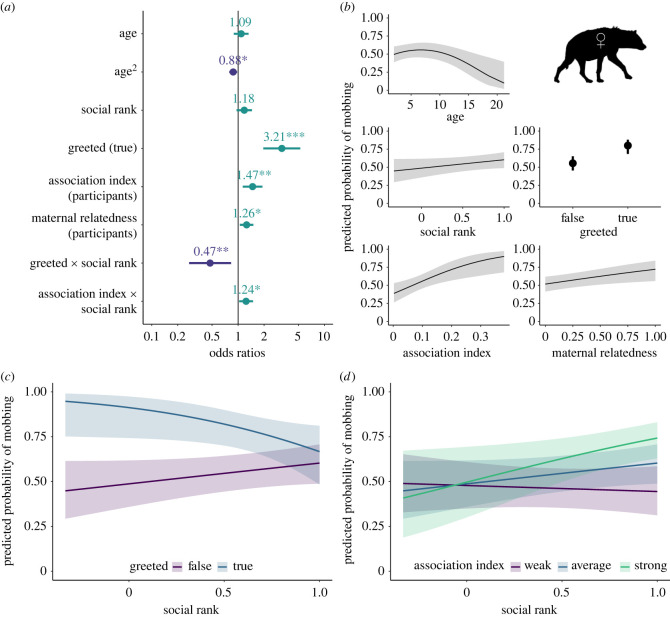
Top model of the predicted probability of mobbing participation by adult female focal hyenas (Model F: *n* focal hyenas = 2280; *n* sessions = 109; *n* mobs = 323; *n* unique hyenas = 169). (*a*). Dots depict coefficient estimates, lines depict 95% confidence intervals, and asterisks depict significance at the following p-values: * = 0.05; ** = 0.01; *** = 0.001. (*b–d*). Lines (or dots) depict estimated marginal means and shaded areas (or vertical lines) depict 95% confidence intervals. (*d*). ‘Weak’ indicates 1 standard deviation below the mean association index, ‘average’ indicates the mean association index, and ‘strong’ indicates 1 standard deviation above the mean association index among hyenas in this dataset.

In our adult female participation model (Model F: *n* = 2280 mob–hyena combinations; figure 4; electronic supplementary material, table S4), focal females that were 6.7 years old (range 2.0–21.2 years) were most likely to mob (OR—age = 1.09, *p* = 0.410; OR—age^2^ = 0.88, *p* = 0.014). Social rank was included in the top model but was not significantly associated with mobbing behaviour (OR—rank = 1.18, *p* = 0.113). Here, as in the model of mobbing occurrence, greetings strongly promoted mobbing behaviour: females that engaged in greeting behaviour during the 5 min before the mobbing event occurred were more likely to mob than those that did not greet (OR—greeted = 3.21, *p* < 0.001). A significant interaction between greeting behaviour and social rank revealed that greeting more strongly promoted mobbing for low- than high-ranking females (OR—greeted × rank = 0.47, *p* = 0.009). Focal females were more likely to mob if other participants were their more frequent associates (OR—association index = 1.47, *p* = 0.004). Again, there was an interaction between frequency of association and social rank: association strength with participants was correlated with higher mobbing probability for high- but not low-ranking individuals (OR—association index × rank = 1.24, *p* = 0.024). Lastly, focal females were more likely to mob if they were related to a larger proportion of the current participants (OR—maternal relatedness = 1.26, *p* = 0.013). Reproductive state was not included in the top model or any model within 6 AIC of the top model.

**Figure 5.**
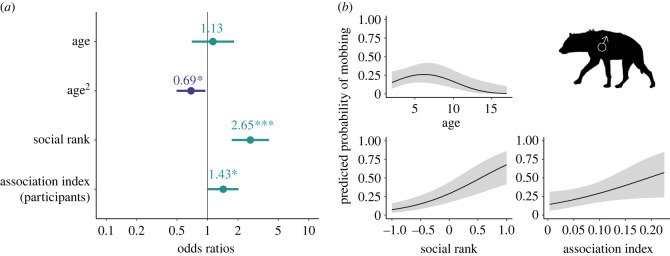
Top model of the predicted probability of mobbing participation by adult male focal hyenas (Model G: *n* focal hyenas = 893; *n* sessions = 101; *n* mobs = 288; *n* unique hyenas = 124). (*a*). Dots depict coefficient estimates, lines depict 95% confidence intervals, and asterisks depict significance at the following *p*-values: * = 0.05; ** = 0.01; *** = 0.001. (*b*). Lines depict estimated marginal means and shaded areas depict 95% confidence intervals.

In our adult male participation model (Model G: *n* = 893 mob–hyena combinations; figure 5; electronic supplementary material, table S4), focal males that were 6.2 years old (range 2.0–16.9 years) were most likely to mob (OR—age = 1.13, *p* = 0.616; OR—age^2^ = 0.69, *p* = 0.025). Higher-ranking males were more likely to mob than their lower-ranking counterparts (OR—rank = 2.65, *p* < 0.001). Focal males that were close associates of the current participants were more likely to participate in that mob than males that were weakly associated (OR—association index = 1.43, *p* = 0.045). Neither dispersal status nor whether the focal hyena greeted during the 5 min before the mob formed were included in the top model or any model within 6 AIC of the top model.

Our participation models revealed that characteristics suggesting a stronger individual, such as being female (the larger sex), prime-aged, and higher-ranking, predicted a higher likelihood of mobbing. Mobbing decisions by females were sensitive to greeting behaviour and to longer-term social factors such as associative relationships, social rank, and kinship. Adult males also participated in mobbing, although less frequently than adult females. Despite having weaker social bonds within the group [69,70], adult male mobbing behaviour was also correlated with variation in social relationships, including dominance rank and association strength. Overall, our results suggest that hyenas' decisions to cooperate in mobbing are strongly affected by the local social environment, including both short-term interactions (greeting) and long-term relationships (association, rank, kinship).

### (c) Who benefits from cooperative mobbing?

To investigate whether mobbing behaviour facilitates the acquisition or defense of food resources, we built a series of logistic models where we modeled the probability of mobbing occurrence, mobbing participation, and benefits to participants as a function of food-related variables such as carcass size and freshness, individual nutritional state, and individual feeding after mobbing events (Models I-L in electronic supplementary material, table S3).

We first inquired whether hyenas are more likely to mob to obtain or defend larger or fresher food resources. Our top model of the occurrence of mobbing at sessions with food (Model I: *n* = 218 sessions; electronic supplementary material, table S4) did not include the term for carcass size but did include the term for carcass freshness (OR—freshness = 0.72, *p* = 0.443), although the effect was non-significant. This suggests that mobs are equally likely to occur across all food sessions, regardless of carcass size or quality.

We next examined whether hyena nutritional state, indicated by individual belly size at the start of the session, affected mobbing participation at carcasses of different sizes. In the model of adult hyena mobbing participation during sessions with food (Model J: *n* = 407 mob–hyena combinations; electronic supplementary material, figure S2 and table S4), a non-significant but negative effect of ‘obese’ belly size suggested that ‘obese’ individuals were perhaps less likely to mob than either ‘fat’ or ‘normal’ individuals (Tukey post-hoc test for belly size: [obese − normal]: HSD = −2.50, *p* = 0.060; [obese − fat]: HSD = −2.54, *p* = 0.057), although there was no difference in mobbing participation between ‘normal’ and ‘fat’ individuals ([fat − normal]: HSD = 0.04, *p* = 0.990). Focal individuals were also less likely to mob at the largest carcasses (Tukey post-hoc test for carcass size: [extra-large − medium]: HSD = −3.75, *p* = 0.027; [extra-large − large]: HSD = −2.63, *p* = 0.048; [large − medium]: HSD = −1.12, *p* = 0.493). Hyenas' age (OR—age = 1.57, *p* = 0.007; OR—age^2^ = 0.83, *p* = 0.033) and social rank (OR—rank = 2.07, *p* < 0.001) also significantly affected their probability of mobbing, as shown in earlier models.

**Figure 6.**
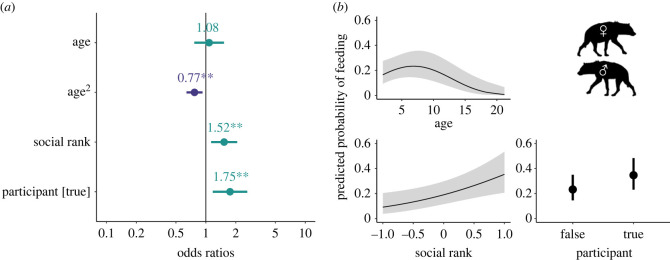
Top model of the predicted probability of the focal hyena feeding during the 5 min immediately after a mob (Model K: *n* focal hyenas = 1049; *n* sessions = 41; *n* unique hyenas = 185). (*a*). Dots depict coefficient estimates, lines depict 95% confidence intervals, and asterisks depict significance at the following p-values: * = 0.05; ** = 0.01; *** = 0.001. (*b*). Lines (or dots) depict estimated marginal means and shaded areas (or vertical lines) depict 95% confidence intervals.

Finally, we inquired whether participants were more likely than non-participants to obtain food after mobbing events. In our model of the probability of adult hyenas feeding in the 5 min after a mob occurred (Model K: *n* = 1049 mob–hyena combinations; figure 6; electronic supplementary material, table S4), focal individuals that mobbed were significantly more likely to feed than individuals that defected (OR—participant = 1.75, *p* = 0.006), even after controlling for age (OR—age = 1.08, *p* = 0.668; OR—age^2^ = 0.77, *p* = 0.005) and social rank (OR—rank = 1.52, *p* = 0.006). However, the model of hyenas feeding at any point during the session (Model L: *n* = 673 session-hyena combinations; electronic supplementary material, table S4) did not include the term for whether or not a focal hyena mobbed during the session, nor did any models within 6 AIC of the top model.

Our results indicate that mobbing increases access to food for spotted hyenas, but that hyenas generally do not adjust their mobbing behaviour based on potential food rewards. One exception to this pattern was that hyenas were less likely to mob at extra-large carcasses such as hippos and elephants; these carcasses last for days in our study area [71], so it may be unnecessary to risk mobbing when simply waiting will yield rewards [45]. Additionally, obese hyenas were less likely to participate in mobbing than thinner individuals, perhaps because they are already satiated or because their obesity may impair their movement (electronic supplementary material, figure S3). Our past research suggested that mobbing increases the probability of any one hyena in the session getting to feed [29]. Here we extend this finding by showing that hyenas who mob during contests with lions over food were more likely to feed in the 5 min after the mob. Although this benefit was short-lived, food obtained by participants during or immediately after mobbing could be substantial, as hyenas can consume enormous quantities of meat extremely quickly [19].

## 4. Conclusion

### (a) Theoretical predictions

Here, our overarching goal was to deepen our understanding of the mediation of collective action in complex societies [72]. Overall, our results support theoretical work suggesting that participants in collective action are often those group members with the lowest cost-benefit ratios [14,15]; individuals in our study who were more likely to mob were also those who were likely to experience more benefits or less risk from mobbing, with characteristics such as being female (the larger sex), prime-aged (for both sexes), and higher-ranking (for both sexes). Interestingly, we also found that participation in mobbing was more sensitive to the potential costs of participation than the potential benefits of success, as hyenas were most likely to mob in sessions where risk was relatively low regardless of potential resource benefits. Studies of mobbing in other species similarly indicate that mobbing participants are often the group members with the lowest cost-benefit ratios [40,73].

Our finding that long-term social ties were associated with mobbing supports theoretical work demonstrating the importance of social network connections to successful group cooperation [16,17], as well as empirical work demonstrating the importance of kinship and social bonds to mobbing behaviour [74,75]. The importance of these long-term social ties (measured here by maternal relatedness and association) also has implications for the theory of fitness interdependence, which suggests that cooperation is promoted when fitness among cooperators is interdependent [76,77]. The most well-established form of fitness interdependence is kin selection [6], but another widespread form arises in systems where individuals form persistent social relationships that are associated with fitness benefits [78,79], as in spotted hyenas [80,81].

Importantly, our work supports a third critical component to successful collective action: short-term prosocial behaviours. Greetings promoted both mob occurrence and participation across age classes, particularly in contexts in which collective action was associated with the greatest risks. These affiliative behaviours could be an important precursor to risky cooperative behaviour via a reciprocal and sequential cooperation strategy (e.g. tit-for-tat) [82], in which an individual bases its decision to cooperate on the behaviour of its partners during prior interactions. The ‘raising the stakes’ model of cooperative investment is one such strategy that has received recent empirical support [83]; in this model, cooperative individuals reduce the risk of exploitation by ‘testing the waters' with low-cost cooperative behaviours before engaging in high-cost cooperation [84]. This ‘water testing’ may occur through low-stakes affiliative behaviours, such as greetings observed here or reciprocal grooming observed in vampire bats [83].

### (b) Why act collectively?

Using a dramatic example of cooperative mobbing against a dangerous predator and competitor, we demonstrate how the coordination of collective action is contextualized within the broader environment of a society characterized by many different types of social relationships. The variation among individuals and relationships in such groups complicates decision-making regarding whether or not to cooperate. However, across contexts, we found that short-term affiliative behaviours boosted individual and group-level cooperative tendencies, sometimes allowing groups with low likelihood of cooperation to nevertheless achieve collective action. The benefits of engaging in this collective action were harder to pin down. Although we found some evidence that individuals gained direct benefits from mobbing lions, we found only mixed support for the prediction that hyenas adjust their mobbing behaviour in response to these potential benefits, and a quarter of mobbing events occurred in the absence of any obvious immediate reward. Overall, we found that, when facing cooperative dilemmas, hyenas, like many other animals living in complex societies [85–87], choose cooperative strategies flexibly and in response to dynamic factors that emerge over multiple time scales [88]. This suggests that social selection may favour individuals that continuously update the social characteristics and relationship value of their group-mates so they can safely navigate risky collective action together.

## Data Availability

The data and source code are available on Dryad [89], as well as on GitHub at https://github.com/tracymont/hyena_mobbing. Supplementary methods and results are provided in electronic supplementary material [90].
